# Smartphone use, wellbeing, and their association in children

**DOI:** 10.1038/s41390-025-04108-8

**Published:** 2025-05-12

**Authors:** Tanja Poulain, Christof Meigen, Wieland Kiess, Mandy Vogel

**Affiliations:** 1 LIFE Leipzig Research Center for Civilization Diseases, Leipzig University, Leipzig, Germany; 2 Department of Women and Child Health, Hospital for Children and Adolescents and Center for Paediatric Research (CPL), Leipzig University, Leipzig, Germany; 3 German Center for Child and Adolescent Health (DZKJ), partner site Leipzig/Dresden, Leipzig, Germany

## Abstract

**Background:**

This study assessed changes in the duration of smartphone use, problematic smartphone use (PSU), quality of life, and their association from 2018 to 2024 in children and adolescents.

**Methods:**

Data were collected between 2018 and 2024 within the LIFE Child cohort study (Germany). We used a repeated cross-sectional dataset containing 2576 data points of 1113 10- to 17-year-old children and adolescents (51% male) who had reported on their quality of life, PSU, and their smartphone use duration. Hierarchical regression analyses were applied to assess associations of PSU, smartphone use duration, and quality of life with the year of assessment and associations of PSU and smartphone use duration with quality of life.

**Results:**

From 2021 onwards, symptoms of PSU and smartphone use durations >3 h/day were significantly more frequent than in 2018. For PSU, these changes were significantly stronger in girls and younger children. Concurrently, quality of life was significantly poorer than in 2018. Both PSU and long smartphone usage durations were significantly associated with lower quality of life.

**Conclusions:**

This study shows the increasingly problematic use of smartphones and its negative association with the overall declining quality of life of children over the last seven years.

**Impact:**

- Problematic smartphone use in children and adolescents has increased in the last seven years, while quality of life has decreased.
- Problematic smartphone use is associated with decreased quality of life, and this association was especially strong in 2022 and 2023.
- In contrast to previous studies, the present time trend analysis covers time periods before and within the Covid-19 pandemic and suggests that the pandemic has reinforced the observed changes in smartphone use and quality of life.
- Restricting children’s smartphone use might help to improve or maintain a higher quality of life.

## Introduction

Healthy development of children and adolescents not only depends on their physical health but also on their mental health and quality of life. Previous studies suggest that in Germany, self-reported health of children has increased^1^ and behavioral difficulties have diminished in the first decade of this century.^2^ In the last few years, however, several social, environmental, and economic crises, especially the Covid-19 pandemic starting in spring 2020, have changed the daily lives of children and adolescents and strained their mental health. Several studies showed that children’s wellbeing and quality of life were significantly poorer in the years 2020 and 2021, when pandemic-related restrictions of daily life were especially strong, than before the pandemic.^3,4^ A recent German study also revealed that the likelihood of poor subjective health, low life satisfaction, and multiple psychosomatic complaints in adolescents has increased significantly from 2017 to 2022.^5^

Another behavior that has also changed significantly in recent years is the use of electronic media, especially smartphones. According to surveys of representative German samples, the percentage of 12- to 18-year-old children owning a smartphone has increased from 62% in 2012^6^ to 94% in 2021.^7^ The time spent with smartphones has also increased considerably in the last years.^8,9^ During the Covid-19 pandemic, especially in the times of school closures and contact restrictions in 2020 and 2021, using the smartphone was often the only way to connect with peers. Therefore, it is not surprising that children and adolescents used smartphones even more frequently in this time period.^10,11^ However, research conducted during the pandemic showed that the use of screen-based media by children and adolescents was associated with frustration and anger^12^ as well as depression, anxiety,^13^ and psychological distress.^11,14^

Children and adolescents use smartphones for several reasons, e.g., communication with others, search for information, self-regulation, but also emotional escape or social avoidance. Independently of these reasons, smartphone use can become problematic. Problematic Smartphone Use (PSU) is defined by excessive use that is accompanied by daily dysfunction and symptoms similar to behavioral addiction, e.g., loss of control or compulsivity.^15,16^ Previous studies showed that both smartphone usage times and symptoms of PSU were more frequent in girls than boys and in older than in younger children.^17–19^ A systematic review from 2019 estimates the prevalence of (clinically relevant) PSU in children and adolescents at 23%.^17^ A review focusing on data collected during the Covid-19 pandemic states a higher estimate of 30%.^20^ Importantly, previous studies (conducted before the Covid-19 pandemic) showed associations between PSU and poorer wellbeing/lower quality of life,^19,21,22^ more behavioral difficulties,^19,23,24^ poorer peer and family relations,^25^ and poor academic performance^26^ in children and adolescents. A meta-analysis on PSU in children and adolescents revealed significant associations with depression, anxiety, perceived stress, and poor sleep (all OR ranging between 1.9 and 3.2).^17^

Limitations of previous studies are the reliance on self-reported data, attrition bias, and missing adjustment for confounding factors.^22^ Studies on recent time trends in quality of life/wellbeing^3–5^ or smartphone use^10,11^ did not investigate whether changes observed at the beginning of the Covid-19 pandemic persisted or attenuated over time. Therefore, one aim of the present project was to assess changes in smartphone use (usage times and symptoms of PSU) and quality of life in children and adolescents from 2018 to 2024, with a specific focus on changes in 2020/21, when pandemic-related cuts in daily life were especially strong, and 2022–2024, when everyday life became more normal again. Based on previous findings, we expected a continuous increase in smartphone use and a decrease in quality of life from 2018 to 2024, with a peak in 2020/2021 that diminished slightly in 2022–2024. Another study aim was to assess the association between smartphone use and quality of life and to determine whether the strength of this association has changed across time. Given the expected increase in smartphone use, we hypothesized that the negative association with quality of life has become stronger in recent years. Finally, we investigated whether time trends in smartphone use or quality of life, and strengths of associations between smartphone use and quality of life differed according to the age and sex of the children. We hypothesized stronger trends/associations in more vulnerable groups, e.g., in younger children and in girls.

## Methods

### Participants

Data were collected between March 2018 and July 2024 within the LIFE Child study, a cohort study conducted at the Leipzig Research Center for Civilization Diseases at Leipzig University (Germany).^27^ The LIFE Child study aims to investigate child development from the prenatal period to young adulthood, with a specific focus on the development of non-communicable physical and mental diseases. All children willing to participate and not suffering from any chromosomal or syndromal disease can participate in the LIFE Child study. They are recruited via advertisement at different health institutions and by word of mouth and mainly come from the city and surrounding areas of Leipzig (Germany). All participating children are invited to a personal (follow-up) visit once a year. During the Covid-19 pandemic, study visits could only take place in periods of lower infection rates. In these time periods, children also went to school.

For the present study, we used a repeated cross-sectional dataset, i.e. each of the participants had participated once or several times. Owning a smartphone, available information on mother’s education, and complete information on PSU and quality of life were preconditions for inclusion in the present study. These conditions were met by 1113 children and adolescents aged 10–17 years. 727 (65%) of these children had participated at more than one time point (308 twice, 419 three or more times), leading to 2576 data points included in the final data set. It was not possible to model individual trends in these children, as only very few participated over the entire period and the starting age of these children varied. Focusing on only one visit per child would have reduced the sample size and thus the statistical power. Therefore, we included all available study visits and accounted for multiple visits in the analyses. Mean age in the final data set was 14.0 years (SD = 2.0). The sex distribution was 49% female (*n* = 1273 data points of 539 girls) and 51% male (*n* = 1303 data point of 574 boys).

The LIFE Child study was designed in accordance with the protocol of Helsinki, and the study program was approved by the Ethics committee of the Medical faculty of Leipzig University (Reg. No. 477/19-ek). All parents provided informed written consent before the inclusion of their children in the study.

### Measurements

#### Problematic smartphone use (PSU)

PSU was assessed using the Smartphone Addiction Proneness Scale.^28^ This scale has been developed in South Korea but its German translation was already used previously,^29^ also within the LIFE Child study.^19,24^ It consists of 15 questions assessing different symptoms of problematic smartphone use, e.g., excessive use, loss of pleasure in other things, or neglect of other activities. Each question can be answered on a 4-point Likert scale ranging from 1 to 4. The item scores were summed up to a PSU total score ranging from 15 to 60, where higher values indicate more problematic use. This score was used for further analysis. Validity and reliability have been checked and approved previously.^28^ The Cronbach’s alpha in this study was 0.84. In a Korean study, a score as high as or higher than 41 was suggested to indicate (clinically relevant) PSU.^30^

### Smartphone use duration

Smartphone use duration was assessed using two questions designed by the authors. They assessed the duration of smartphone use on a typical school day or a weekend day. The response options to these two questions changed at the beginning of 2021. Therefore, we only compared smartphone usage times that could be distinguished in both versions. All response options below 3 hours per day (old version: response categories “1–2 h” and lower, new version: response categories “2.5 h” and lower) were summarized to <3 h/day (low) while all response options as high as 3 h per day or more (old version “3–4 h” and higher, new version: “3 h” and higher) were summarized to ≥3 h/day (high). Due to the high association between usage times on weekends and weekdays, we only used usage time on weekends for further analysis.

### Quality of life

The KIDSCREEN-27^31^ was used to assess participants’ quality of life. The questionnaire assesses five dimensions of quality of life (physical wellbeing, psychological wellbeing, satisfaction with peer relationships, with parent relationship and parental support, and with school environment) using a total of 27 items. Each question is answered on a 5-level Likert scale. The item scores on each dimension are summarized to sum scores, which are subsequently transformed to sex-specific t-scores (mean = 50, sd = 10). In the present study, the t-scores of the 5 dimensions were averaged to a total quality of life score. Cronbach’s alpha for this total score was 0.92.

### Covariates

Age, sex, and the mothers’ education were considered as covariates. Education is an indicator of socio-economic status, which has been shown to be associated with both children’s smartphone use^19,32^ and their quality of life.^33^ For the assessment of education, information on mothers‘ school and professional education (two items in a parental questionnaire) were transformed to a score ranging between 1 and 7, based on a procedure applied in a large German population-based study.^34^ Higher values indicate higher education.

### Data analysis

Data were analyzed using R.^35^ Data were described in terms of means and standard deviations (for continuous variables) or numbers and percentages (for categorical variables). In a first step, we assessed associations between PSU total score or smartphone use duration on weekends (as dependent variables) and year of assessment (2018–2024, independent variable, reference = 2018). Age (continuous), sex, and maternal education were included as covariates. Furthermore, we checked whether these associations were moderated by the age or sex of the child.

In a second step, we assessed associations between quality of life (as dependent variable) and year of assessment, PSU total score, and smartphone use duration (as independent variables), adjusting for child age, sex, and maternal education. We then checked whether associations between quality of life and PSU total score or smartphone use duration were moderated by year of assessment, age, or sex.

All analyses were assessed using hierarchical linear models (for continuous dependent variables) or hierarchical generalized linear models (for dichotomous dependent variables), with the participants’ ID included as random factor.

## Results

Characteristics of the study sample are shown in Table 1. The average quality of life t-score was 52.4 (sd = 7.3), which is slightly higher than the expected mean of 50. The average score of mothers’ education (possible range 1–7) was 5.3 (sd = 1.5), indicating a rather high education in participants’ families.

**Table 1:** Characteristics of the study population (*n* = 2576 data points of 1113 children)

Variable		
Sociodemographics		
Age	Mean (sd)	14.0 (2.0)
Sex	*n* (%) male	1303 (51%)
	*n* (%) female	1273 (49%)
Education mother	mean (sd)	5.3 (1.5)
Year of assessment	*n* (%) 2018	351 (14%)
	*n* (%) 2019	510 (20%)
	*n* (%) 2020	238 (9%)
	*n* (%) 2021	344 (13%)
	*n* (%) 2022	420 (16%)
	*n* (%) 2023	463 (18%)
	*n* (%) 2024	250 (10%)
Media use		
PSU total score	Mean (sd)	28.6 (6.6)
(Clinically relevant) PSU	*n* (%)	56 (3%)
Smartphone use duration on weekends	*n* (%) < 3 hours/day	1245 (48%)
	*n* (%) ≥ 3 hours/day	1331 (52%)
Quality of life		
Quality of life total score	Mean (sd)	52.4 (7.3)

Children not owning a smartphone (*n* = 110 data points) were excluded from analyses. As revealed by two-sample t-tests or chi-squared tests, these children were significantly younger (mean age = 11.6), were more frequently male (66%), had mothers with a higher education (mean = 5.9), and reported a higher quality of life (mean = 54.5) than children owning a smartphone, i.e., than those included in our analyses.

### Associations of PSU, smartphone use duration, and quality of life with year of assessment

Regarding the PSU total scores, the analyses revealed significantly higher scores in 2021 (estimated mean = 28.5), 2022 (estimated mean = 29.2), 2023 (estimated mean = 30.0), and 2024 (estimated mean = 29.9) compared to 2018 (estimated mean = 27.6). The betas and *p*-values for these differences are shown in Table 2.

**Table 2:** Differences in PSU, smartphone use duration, and quality of life from 2019 to 2024 compared to 2018

	PSU		Smartphone use duration >3 h/day		Quality of life	
Year	b (95% CI)	*p*	OR (95% CI)	*p*	b (95% CI)	*p*
2018	Reference					
2019	0.32 (−0.34, 0.97)	0.347	0.95 (0.58, 1.25)	0.414	−0.26 (−0.96, 0.45)	0.478
2020	−0.09 (−0.92, 0.74)	0.837	1.15 (0.71, 1.85)	0.573	−0.21 (−1.10, 0.69)	0.649
2021	0.94 (0.17, 1.74)^a^	0.017	2.42 (1.43, 3.49)	<0.001	−1.34 (−2.18, −0.51)	0.002
2022	1.60 (0.82, 2.38)^a^	<0.001	2.62 (1.70, 4.05)	<0.001	−2.05 (−2.89, −1.20) ^b^	<0.001
2023	2.42 (1.61, 3.23)^a,b^	<0.001	3.15 (2.03, 4.89)	<0.001	−2.35 (−3.23, −1.47) ^b^	<0.001
2024	2.34 (1.39, 3.29)	<0.001	4.38 (2.59, 7.40)	<0.001	−2.80 (−3.83, −1.77) ^b^	<0.001

^a^Significant interaction with age: effects stronger in younger children

^b^Significant interaction with sex : effects stronger in girls than boys

**Fig. 1:**
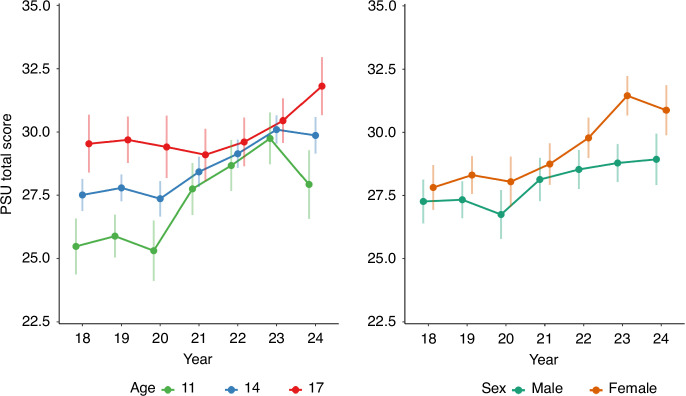
Changes in PSU total scores from 2018 to 2024 by age (left) and sex (right). Changes were stronger in younger children and in girls. For better visibility, we only illustrate three age groups (11-, 14-, and 17-year-olds).

As shown by significant interactions with age, the increase in PSU total scores from 2018 to 2021, 2022, and 2023 became significantly weaker with increasing child age (beta = −0.45, −0.52, −0.56, *p* = 0.031, 0.014, and 0.008, respectively). In addition, a significant interaction with sex (*p* = 0.005) revealed that the observed increase in PSU from 2018 to 2023 was significantly stronger in girls (beta = 3.01, *p* < 0.001) than in boys (beta = 1.81, *p* = 0.001). The changes in PSU total scores across time are illustrated in Fig. 1, stratified by age and sex.

Interestingly, Fig. 1 also indicates a decrease in PSU scores from 2023 to 2024 in 11-year-olds. A supplementary analysis revealed that this decrease (from PSU total score 31.2 to PSU total score 27.0) was statistically signifiant (*p* = 0.004). Such a significant decrease was not observable in any other age group.

Similar to the results regarding PSU total scores, the frequency of children showing smartphone use durations ≥3 h per weekend day was significantly higher in 2021 (estimate = 57%), 2022 (estimate = 61%), 2023 (estimate = 66%), and 2024 (estimate = 73%) than in 2018 (estimate = 37%). The odds ratios and p-values are shown in Table 2. In contrast to what was observed for PSU, changes in smartphone use durations did not differ significantly depending on age or sex.

**Fig. 2:**
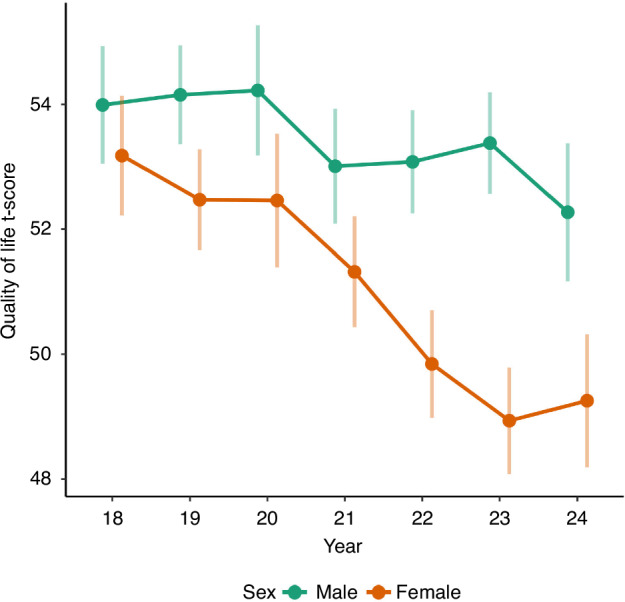
Changes in quality of life from 2018 to 2024 by sex. The decrease in quality of life was stronger in girls than in boys.

Regarding quality of life, the analyses revealed a significantly lower quality of life in 2021 (estimated mean = 52.2), 2022 (estimated mean = 51.5), 2023 (estimated mean = 51.2), and 2024 (estimated mean = 50.8) than in 2018 (estimated mean = 53.6). Betas and *p*-values are shown in Table 2. The strengths of changes were not significantly moderated by age. However, significant interactions with sex (*p* = 0.003, <0.001, and 0.023, respectively) revealed that the changes in quality of life between 2018 and 2022, 2023, and 2024 were significantly stronger in girls (beta = −2.99, −3.73, and −3.28, all *p* < 0.001) than in boys (beta = −1.26, −1.05, and −2.22, *p* = 0.039, 0.096, and 0.004, respectively). Figure 2 shows the changes in quality of life across time, stratified by sex.

### Association of quality of life with PSU and smartphone use duration

Higher PSU total scores were significantly associated with lower quality of life (b = −0.38, *p* < 0.001). Significant interactions with year of assessment (*p* = 0.015 and 0.049) revealed that this association was significantly stronger in 2022 (b = −0.55, *p* < 0.001) and 2023 (b = −0.46, *p* < 0.001) than in 2018 (b = −0.40, *p* < 0.001). The association between quality of life and PSU was not signifiantly moderated by age or sex.

**Fig. 3:**
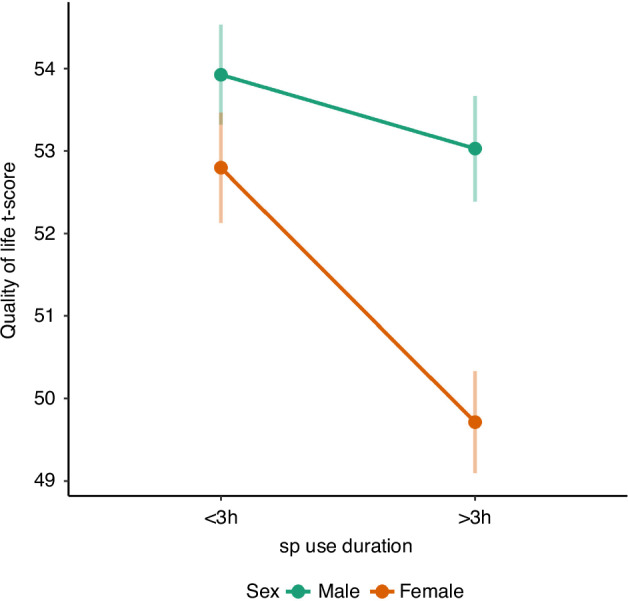
Association between smartphone use duration and quality of life by sex. The association was stronger in girls than in boys.

Similar to PSU scores, smartphone use durations ≥3 h/day were associated with significantly lower quality of life (b = −1.93, *p* < 0.001). This association was not moderated by year of assessment or child age. However, a significant interaction with child sex (*p* < 0.001) showed that the association between decreased quality of life and long smartphone usage times was stronger in girls (b = −2.53, *p* < 0.001) than in boys (b = −1.26, *p* < 0.001). The interaction between the duration of smartphone use and sex is also shown in Fig. 3.

## Discussion

### Main findings

The aim of this project was to investigate changes in children’s and adolescents‘ smartphone use and quality of life within the last seven years. Overall, most participants in the present study reported long smartphone usage times (>3 h/day). The prevalence of (clinically relevant) PSU, however, was small (3%) compared to estimates stated in previous reviews.^17,20^ As hypothesized, the duration of smartphone use and symptoms of PSU increased significantly from 2018 to 2024, while quality of life decreased in the same time period. In line with our expectation, these changes first became statistically significant in 2021, i.e., during the Covid-19 pandemic. With the exception of PSU-related changes in 11-year-olds, we found no evidence for a trend back to normality at the end of/after the pandemic. Therefore, the hypothesis of an overall trend back to normality could not be confirmed.

Another study aim was to explore (time changes in) the association between quality of life and smartphone use. As expected, quality of life showed significant negative associations with PSU and smartphone use duration. Also in line with our hypotheses, the association between quality of life and PSU was significantly stronger in 2022 and 2023 than in 2018. Finally, the analyses revealed that time trends and associations were (in some cases) stronger in girls and younger children, as expected.

### Time trends in smartphone use

Our results show that smartphones have become more and more indispensable in the lives of children and adolescents. However, not only the time spent with the smartphone has increased in the last years; smartphone use has also become more problematic. Importantly, the increase in smartphone use and PSU was especially strong in the last four years (2021–2024 compared to 2018). Therefore, one might suggest that this increase is due to the Covid-19 pandemic. This suggestion is supported by previous studies showing increased smartphone use at the beginning of the pandemic compared to the time before.^10,11^ During Covid-19-related school closures (especially in 2020 and 2021), electronic devices, including smartphones, took on special importance as they allowed children to stay in virtual contact with others, to complete schoolwork from home, and to relieve boredom. Even though children’s daily lives became more normal again in 2022, the smartphone may not have lost its importance, but rather remained an integral part of children’s everyday life. The increase in (problematic) smartphone use could also be explained by new apps and developments on the internet that increase usage time, e.g., (videos on) personalized feeds.

### Trends in quality of life and associations with smartphone use

While smartphone use increased and became more problematic from 2018 to 2024, quality of life declined in the same time period. Again, one reason might be the Covid-19 pandemic that disturbed children’s everyday life.^3,4^ In addition, other events such as the climate and energy crisis and the Russian-Ukrainian war are worrying children and adolescents and might impact their quality of life.

Another finding of the present study is that quality of life was significantly associated with longer smartphone usage times and more symptoms of PSU. This result is in line with previous research findings.^17,19,21^ Interestingly, we showed that the association between PSU and quality of life was especially strong in 2022 and 2023. This finding might indicate that in times where the smartphone gains more and more importance in the lives of children and adolescents, its link with wellbeing and quality of life also increases.

Importantly, our results do not allow us to draw any conclusions about whether smartphone use influences quality of life or vice versa. Children with a lower quality of life may use their smartphone for escape or relaxation, and excessive smartphone use might (further) increase psychological distress.^21^ It is also important to emphasize that quality of life includes various areas such as mental and physical health, but also satisfaction with family, school and peers. It is possible that all aspects (including smartphone use) influence each other and are also influenced by other parameters that were not taken into account in this study.

### Differences depending on child age and sex

The time trends and associations between quality of life and smartphone use partly differed depending on child age and sex. Regarding the age of children, the increase in PSU between 2018 and 2022, 2023, and 2024 was stronger in younger than in older children. While younger children reported fewer PSU symptoms than older children in the years 2018–2020, there was hardly any difference in age in the years 2021–2023. In particularly challenging situations such as the Covid-19 pandemic, younger children may be more likely to seek refuge in electronic media, including smartphones. Interestingly, the youngest children in our sample (11-year-olds) were also the only ones for whom symptoms of PSU decreased again significantly between 2023 and 2024. Young children may not only react more intensely to challenging situations but also – in a positive sense – to a normalization after a challenging time period.

Regarding child sex, our analyses showed that the increase in PSU from 2018 to 2023 and the decline in quality of life between 2018 and 2022–2024 were stronger in girls than in boys. These findings might indicate that girls suffered more from changes associated with the Covid-19 pandemic than boys, as shown in previous studies.^36,37^ Importantly, the association between smartphone use duration and quality of life was also stronger in girls than in boys. In line with previous findings,^38,39^ this result suggests that effects of smartphone use on quality of life and/or vice versa might be particularly strong in girls. This sex difference might be explained by differences in the activities girls and boys perform on their smartphones. Girls have been shown to use social media more frequently than boys,^38^ and social media use has been shown to be associated with internalizing problems and poorer mental health in adolecents.^40,41^

Taken together, our findings suggest that younger children and girls are vulnerable groups whose smartphone behavior and, in the case of girls, quality of life change more easily due to challenging situations (e.g., a pandemic).

### Strengths and limitations

This study investigated time trends in quality of life and smartphone use in a large sample of healthy children and adolescents across a long time period including the Covid-19 pandemic. Furthermore, both smartphone usage time and symptoms of PSU were assessed. Limitations concern the under-representation of children from families with lower education and the reliance on self-reported data. Especially recalls of own media usage times can be inaccurate or biased. Furthermore, the questionnaire for the assessment of the smartphone use duration had changed during the survey period. Even if response categories were summarized accordingly, an influence on the children’s responses cannot be ruled out. Finally, this is a cross-sectional study. Therefore, we cannot draw conclusions on possible causalities between smartphone use and quality of life.

Future studies may investigate to what extent the trends in quality of life, smartphone use, PSU, and their inter-relationship will continue in the coming decades. In addition, it might be interesting to investigate trends and relationships in other pediatric cohorts, e.g. children suffering from various neurodevelopmental or mental health disorders, e.g., attention deficit hyperactivity disorder or depression.

## Conclusion

The results of the study show that the use of smartphones has become increasingly problematic over the past seven years and is increasingly associated with the overall declining quality of life of children and adolescents. These findings indicate a strong link between smartphone use and quality of life. They underline the importance of strengthening children’s and adolescents’ quality of life and helping them to limit the time they spend on their smartphones, e.g., by offering alternative activities, setting time limits, or creating a suitable family environment. As children spend a large part of their day at school, school would be a possible place where health literacy, including media education, could be taught^42^ and where time restrictions could be implemented.

## Data Availability

The datasets generated and/or analyzed during the current study are not publicly available due to ethical restrictions. The LIFE Child study is a study collecting potentially sensitive information. Publishing data sets is not covered by the informed consent provided by the study participants. Furthermore, the data protection concept of LIFE requests that all (external as well as internal) researchers interested in accessing data sign a project agreement. Researchers that are interested in accessing and analyzing data collected in the LIFE Child study may contact the data use and access committee (forschungsdaten@medizin.uni-leipzig.de).
